# A minireview of hydroamination catalysis: alkene and alkyne substrate selective, metal complex design

**DOI:** 10.1186/s13065-019-0606-7

**Published:** 2019-07-11

**Authors:** Jingpei Huo, Guozhang He, Weilan Chen, Xiaohong Hu, Qianjun Deng, Dongchu Chen

**Affiliations:** Institute of Electrochemical Corrosion, College of Materials Science and Energy Engineering, Foshan University, Foshan, 528000 People’s Republic of China

**Keywords:** Hydroamination, Atom economy, C–N bond, Metal catalysis

## Abstract

Organic compounds that contain nitrogen are very important intermediates in pharmaceutical and chemical industry. Hydroamination is the reaction that can form *C*–*N* bond with high atom economy. The research progress in metals catalyzed hydroamination of alkenes and alkynes from the perspective of reaction mechanism is categorized and summarized.
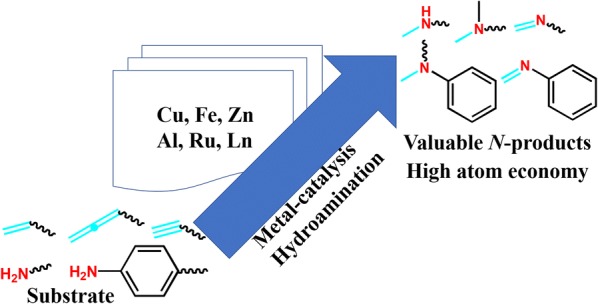

## Introduction

More and more attention are attracted in hydroamination reaction as a tool for *N*–*H* synthesis, plenty of complementary synthetic methods have come to the fore for the development of intensified and industrially relevant *C*–*N* forming processes [1, 2]. According to our statistics, synthesizing *C*–*N* via hydroamination reaction has become a promising area of research, experiencing growing diversification [3, 4]. Since the first publications on this hydroamination reaction over past years, close to 450 research papers have been published on this topic [5, 6]. Further statistics indicate 78% of the published research can be classified as substrates which work under mild conditions as follows [7, 8]. At the same time, with the development of hydroamination, various catalytic systems have been gradually systematized, and many breakthrough progresses have been made [9, 10].

The attractive and challenging methods for the formation of *C*–*N* bonds are hydroamination reactions. In this review, we will mostly focus on recent developments in the effects of different substrates containing *N*–*H*. In the meantime, usage of the term hydroamidation is not only including the substrate classes of saturated fat primary amine, saturated fatty secondary amine and unsaturated fatty amine, but extended to structurally related compounds with selective, reactivity and productive yield. Moreover, intra- and inter-molecular hydroamination reactions will be mentioned as well if they are necessary for the discussion or might act as springboard for future research.

## The effects of different compounds containing *N*–*H*

### Saturated fatty primary amine

Fatty primary amines (C_1_ to C_12_) are essential intermediates for the chemical and pharmaceutical industries. A large amount of fatty primary amine and the corresponding derivatives are according to their cationic surface activity.

**Scheme 1:**
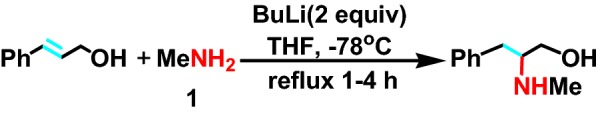
Addition of primary amines to cinnamyl alcohol

In 2007, Barry et al [11] introduce organolithium into the hydroamination reaction between the molecules of cinnamyl alcohol and primary amine **1** (Scheme 1). In the presence of metallic lithium, the nonterminal olefin and primary amine compounds were acquired, such as methylamine, benzyl amine butyl amine, but the yield is only about 50%. On the one hand, it can undergo a favorable proton transfer process to give the more stable amido-alkoxide, thus shifting the equilibrium in the desired sense. It is found that they do not introduce carbonyl and halogenated compounds to saturated fats. Besides, the reaction conditions are very hard, and the reaction temperature needs to drop to − 78 °C.

**Scheme 2:**
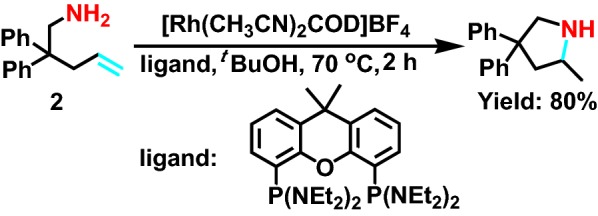
Rh-catalyzed hydroamination of primary aminoalkenes

**Scheme 3:**
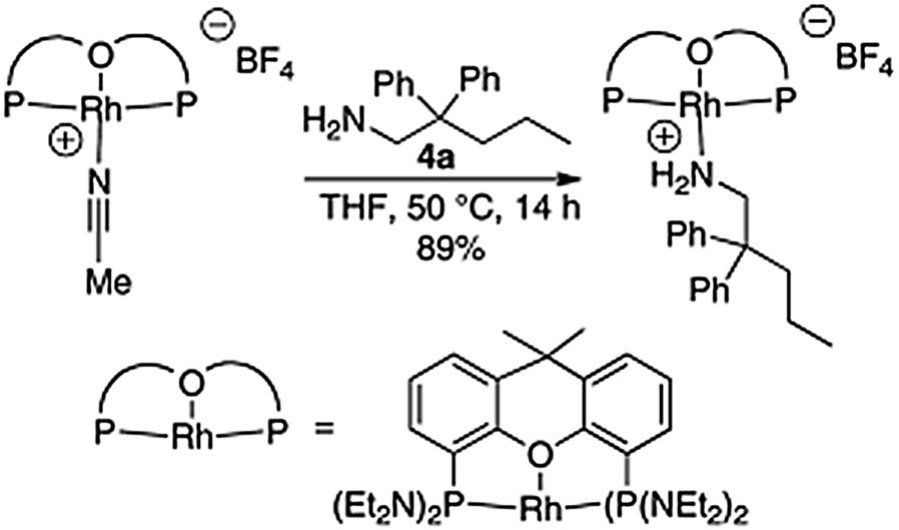
The forming Rh complex intermediate state of hydroamination reaction

[Rh(CH_3_CN)_2_COD]BF_4_ possesses a great deal of benefits, including high activity and effective. In 2010, Julian et al [12] use this compound to catalyze the intramolecular hydroamination reaction (Scheme 2). The catalyst has strong applicability, and it can achieve very high catalytic effect, whether it has chlorine atom (Cl), ester base (COO), ketone (CO), nitrile (CN), or hydroxyl (OH) without protection. Meanwhile, this rhodium ligand is undefined from the ligand, which is formed by the late transition metal such as palladium (Pd), platinum (Pt), iridium (Ir), after the rhodium ligand and the carbon-carbon double bond on the bottom of primary amine substrate **2** formed complexes, it will not reverse, as a result of competitive catalyst decomposition, forming a non-cyclic precursor, and greatly improving the efficiency of molecular hydrogen amination. Besides, the forming Rh complex of hydroamination reaction was given in the Scheme 3.

**Scheme 4:**
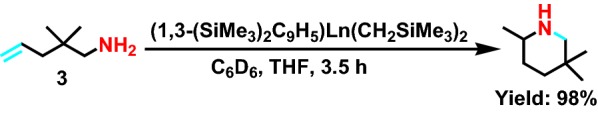
Hydroamination of 2,2-dimethylpent-4-enylamine catalyzed by (1,3-(SiMe_3_)_2_C_9_H_5_)Sc(CH_2_SiMe_3_)_2_(THF)

In 2010, Xu group [13] firstly take advantage of Ln(CH_2_SiMe_3_)_3_(THF)_2_ and Indenyl with half-Sandwich *η*^5^ ligands, separating the catalyst and determine its structure via crystal diffraction. The experiment demonstrates that the catalyst is very effective for the intramolecular hydroamination synthesis of nitrogen heterocyclic compounds. As for the *C*_6_*D*_6_ solvent, the intramolecular hydroamination reaction was found in saturated fatty primary amine substrate **3** (Scheme 4). Consequently, these ligands containing yttrium and dysprosium, are highly active in a series of saturated fatty primary amine substrates, and are relatively easy to form nitrogen heterocyclic compounds (yielding 98%).

**Scheme 5:**
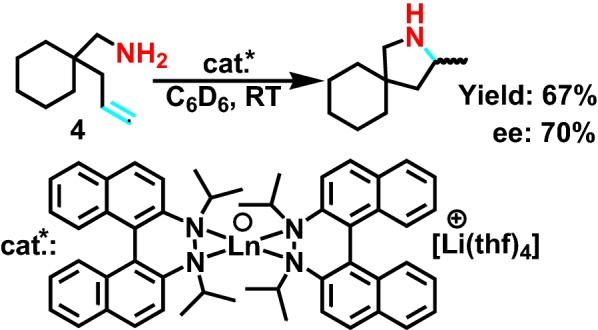
Hydroamination/cyclization of 1-(aminomethyl)-1-allylcyclohexane by Li_2_[(R)-C_20_H_12_N_2_-(C_10_H_22_)]

2005, Collin et al [14] reported the lanthanide compounds catalyst intramolecular asymmetric hydroamination reaction of saturated fatty primary amine **4** (Scheme 5), which has undergone the activation of isopropyl group, and further obtained spiral pyrrolidine. The selectivity of the reaction is good, and the *e.e*. value reaches 70%.

**Scheme 6:**
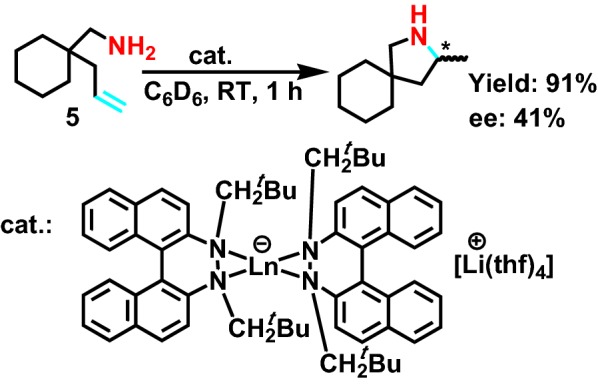
Hydroamination-cyclization of 1-(aminomethyl)-1-allylcyclohexane by {Li(THF)_4_}{Ln[(R)-C_20_H_12_N_2_(C_10_H_22_)]_2_}

While Collin group [15] designed a kind of highly active lanthanide to catalyze intramolecular asymmetric hydroamination reaction of saturated fatty primary amine **5** (Scheme 6), introducing the tertiary butyl group into the catalyst, it can get high yield of secondary amine derivatives, the maximum yield can reach 94%, and it has the very good stereoselectivity, the *e.e*. value reaches 40%.

**Scheme 7:**
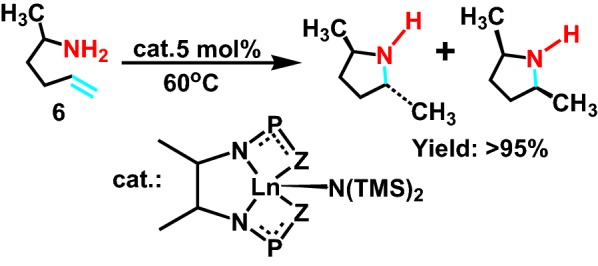
Catalyzed cyclization of 2-aminohex-5-ene

In 2003, Kim et al [16] formed a bident ligand through lanthanide and triphenylphosphine, which catalyzed intramolecular hydroamination reaction of saturated fatty primary amine **6** (Scheme 7), and synthesized a variety of secondary amines. But the selectivity of this reaction is not so good, vice product was generated. In addition, the study found that compared with the covalent radius of neodymium and yttrium, the covalent radius of dysprosium is small. Therefore, when it catalyzes intramolecular hydroamination reaction, it can make the product do not change the configuration in a short time, and further raise the antipodal selectivity.

**Scheme 8:**
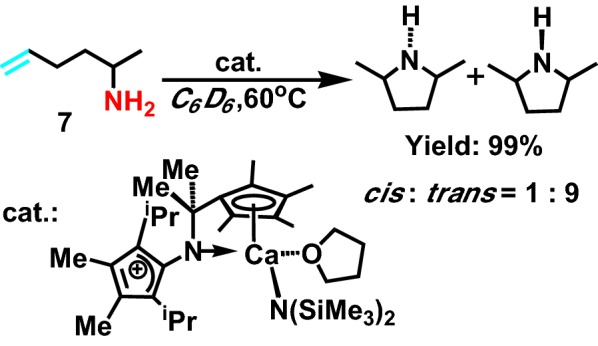
Hydroamination reaction of terminal aminoalkenes and alkynes catalyzed by Ca catalyst

In 2008, aiming to synthesizing a novel kind of ligand, Tamm group [17] select rare earth metals and alkali metal as the hydroamination reaction catalyst, limiting the geometry of that catalyst. And then its structure was determined by single crystal diffraction. Maybe due to the catalyst is meso-structure, consist of two cyclopentadiene group, exhibiting strong ability of electron-donating. It also greatly enhances the activity of intramolecular hydroamination reaction of saturated fatty primary amine **7** (Scheme 8), beneficial for shifting from *trans* to *cis*.

### Saturated fatty secondary amine

Nitrogen compounds are widespread in many natural organic compounds and possess a series of physiological activity [18, 19]. After pharmacology studies, these compounds have good anti-inflammatory effects such as antiseptic, antifungal and other aspects [20]. Therefore, the reaction of hydroamination has been one of the hotspots in the research of organic synthesis [21]. In order to further enrich the kinds of nitrogenous compounds, chemists synthesized a variety of multifunctional nitrogen compounds [22]. Based on saturated fatty secondary amine, it will show more complex molecular structure as well, meeting the needs of pharmaceutical industry [23].

**Scheme 9:**
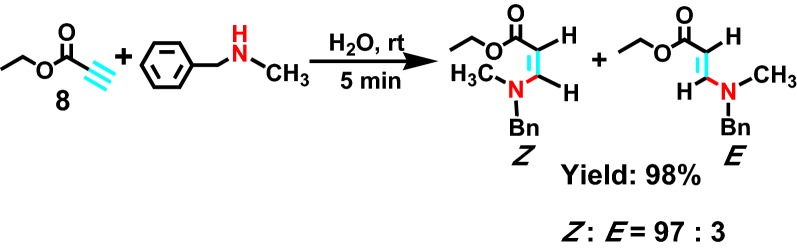
Reactions of thiols and amines with ethyl propiolate

In 2010, Randive et al [24] found that it is good for the intermolecular hydroamination reaction in water phase. As for propiolic acid ethyl ester and saturated fat secondary amine **8** (Scheme 9), including dimethylamine, diisopropyl amine and piperidine, sequentially beta amino ester compounds were acquired. This reaction not only has high regio-selectivity and stereo-selectivity, using the green and inexpensive solvent, providing a pioneering research method for studying the hydroamination reaction.

**Scheme 10:**
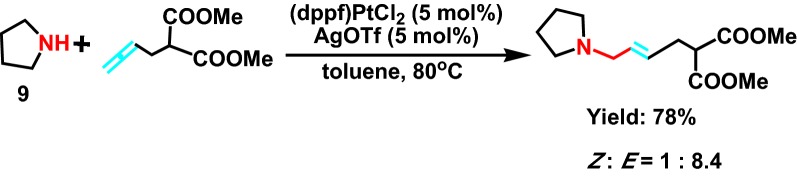
Intermolecular hydroamination of monosubstituted allenes with secondary alkylamines catalyzed by a mixture of (dppf)PtCl_2_ and AgOTf in toluene at 80 °C

**Scheme 11:**
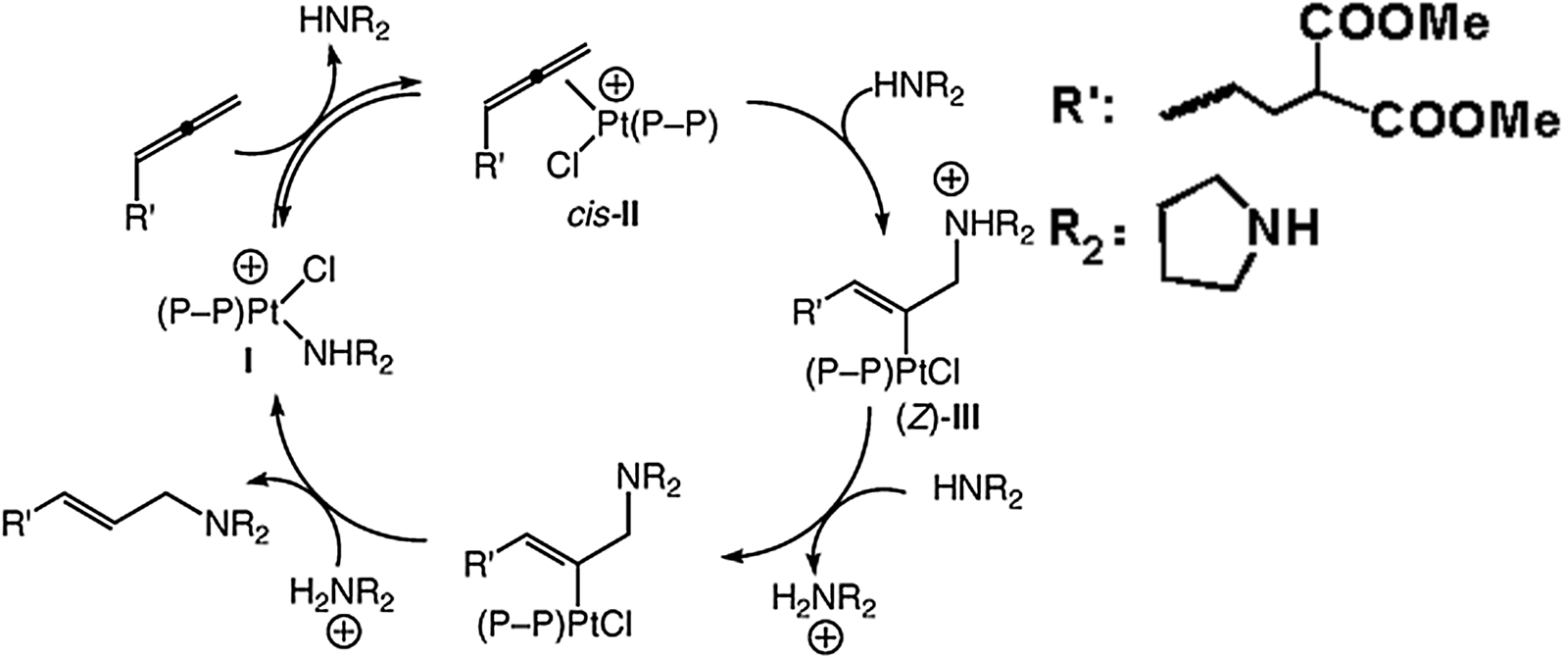
A mechanism for the platinum catalyzed hydroamination of allenes with secondary alkylamines

In 2010, Toups and Widenhoefer [25] co-found a new intramolecular palladium catalyzed hydroamination reaction with substrate **9** (Scheme 10) and divinyl. It is involved that this reaction is initiated by the oxidation reaction between allyl group of propadiene and the silver trifluoromethane. palladium ion (Pd^2+^) attack from the back of the propadiene, forming the π propadiene ligand of cationic palladium, and generating the *trans* product at last, and the corresponding reaction mechanism was displayed (Scheme 11).

**Scheme 12:**
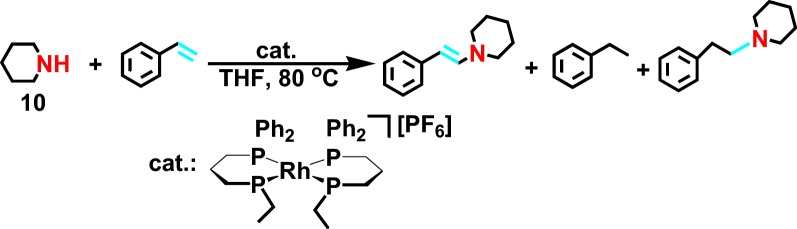
Rh-catalyzed hydroamination of styrene with piperidine

In 2010, Jimenez et al [26] reported the hydroamination based on the *N*-substrate **10** (Scheme 12) with intermolecular regio-selectivity catalyzed by Rh^+^ salt, producing *anti*-Markovnikov products. In addition, the structure of the catalyst was confirmed by single crystal diffraction. It was found that Rh^+^ and diphenylphosphine can generate trans chelate, greatly promoting the formation of *anti*-Markovnikov products. However, this reaction has some limitations. This reaction limited to saturated fatty secondary amine and produced a large number of by-products.

**Scheme 13:**
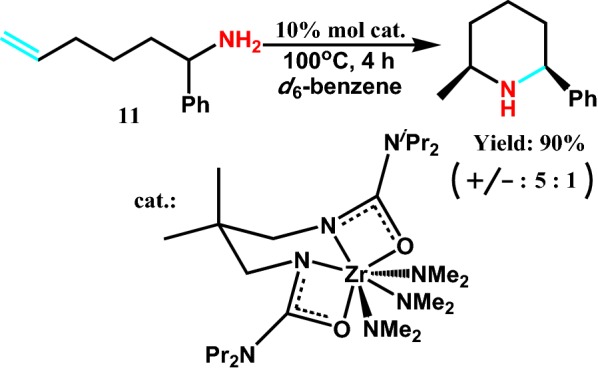
In situ catalyst screening of both primary and secondary aminoalkene substrates

In 2009, Leitch et al [27] reported intramolecular hydroamination reaction of saturated fatty secondary amine catalyzed by Zr(NMe_2_)_4_ proligand. This method is used to synthesize six nitrogen heterocyclic synthesis of various kinds of activity in different substituted allyl amines **11** (Scheme 13), but also are applied for synthesizing natural product intermediates. More importantly, Zr(NMe_2_)_4_ have high chemical selectivity for saturated fatty secondary amines. It is unnecessary for shape didentate ligands in the process of ring forming.

**Scheme 14:**
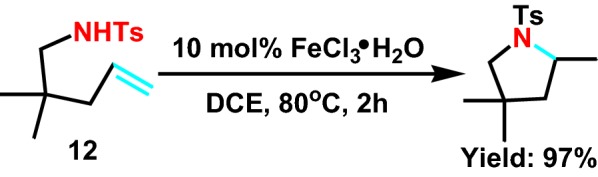
Intramolecular hydroamination of amino olefins by FeCl_3_·6H_2_O

Exploiting more practical, less limitations of catalyst are used for intramolecular hydroamination, in favor of seeking another new scheme. With the direction of Komeyam et al [28] they studied a simple and effective method for hydroamination with **12** (Scheme 14), and synthesized pyrrole derivatives under the catalysis of ferric chloride through intramolecular hydrogenation amination. The reaction conditions are moderate, regardless of any ligands.

**Scheme 15:**
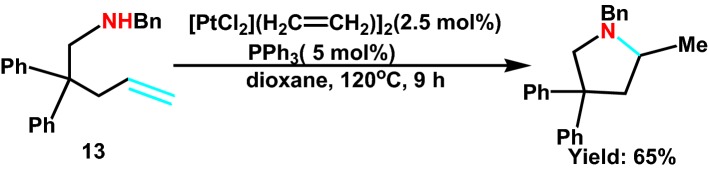
Hydroamination of amino olefins catalyzed by a mixture of [PtCl_2_(H_2_CdCH_2_)]_2_ and PPh_3_ in Dioxane at 120 °C

In 2005, Bender and Widenhoefer [29] jointly designed the intramolecular amination of saturated fatty secondary amine **13** (Scheme 15). The substrate, such as gamma aminolefine, was induced by the Pt-based catalytic system, and the corresponding five membered nitrogen heterocyclic compounds were obtained. The author speculated that the formation of trans Pt-C bond by platinum hydride, is conducive to the deprotonation and get good yield.

**Scheme 16:**
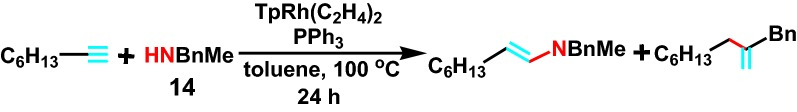
TpRh(C_2_H_4_)_2_/PPh_3_-catalyzed hydroamination of 1-octyne with amines

Fukumoto research group [30] in 2007 designed and synthesized organic rhodium catalyst to catalyze the hydroamination between the alkyne and saturated fatty secondary amine **14** (Scheme 16). For the intermolecular alkynes hydroamination synthesis of the corresponding *anti*-Markovnikov enamines and imines, the organic rhodium metal catalyst has good regio-selectivity. The author also explains the result of this specificity, because the metal rhodium complex can not turn over after its coordination with the unsaturated bond.

**Scheme 17:**
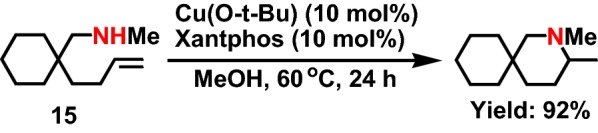
Cu(I)-catalyzed intramolecular hydroamination of aminoalkene

In 2009, Ohmiya et al [31] reported the synthesis of pyrrolidine and piperidine derivatives by intramolecular hydroamination of terminal olefin catalyzed by copper. The experiment showed that the Cu complex could effectively catalyze the hydroamination reaction of saturated fatty secondary amine **15** (Scheme 17). After introducing methoxy group (–CH_3_), fluorine atom (F), nitrile group (–CN) and ester group (–COO) on the amine group, the cyclization process was not affected, and the yield was very high at the same time. It is worth noticing that the mechanism of the phenomenon is explained by the authors. The carbon carbon double bonds on olefin and alkyl copper formed copper olefin-*π* ligands. Because of the protonation effect, the copper ligand on the five membered rings eliminated faster than the beta hydrogen, and finally formed the enamine and hydrides of copper.

**Scheme 18:**
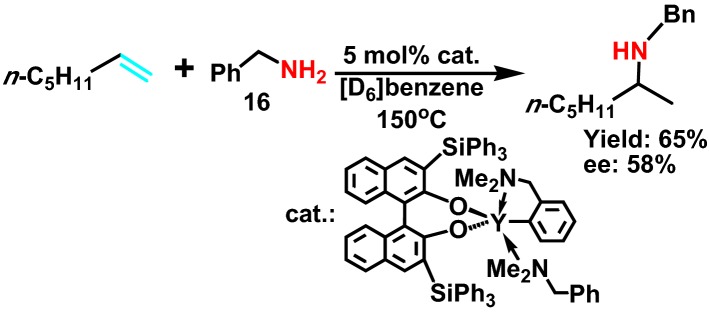
Asymmetric intermolecular hydroamination of 1-alkenes with a primary amine

In 2010, Reznichenko et al [32] reported the asymmetric hydroamination reaction catalyzed by several lanthanide catalysts. Chain olefins, such as 1-heptene and benzyl amines **16** (Scheme 18), has very high and selective enantioselectivity in hydroamination, and has little *by*-product. This method often produces chiral amine ligands in the reaction process. Research shows that even when para benzyl amines have a methoxy, it will greatly reduce the asymmetric hydroamination activity of chain olefin.

**Scheme 19:**
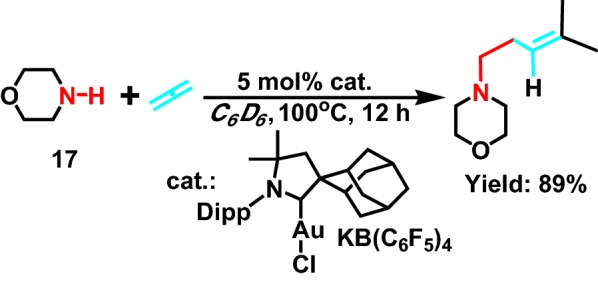
Hydroamination of allenes with secondary alkylamines

Kang et al [33] reported in 2006 that the intermolecular hydroamination between allene and saturated fatty secondary amine **17** (Scheme 19) catalyzed by Au. In the process of this reaction, Au^+^ substrate formed carbene ligand and produced a chiral center, ultimately contributing to the formation of a single markovnikov product. This gold complex has been proved to be a highly efficient catalyst for the hydroamination between saturated fatty secondary amine and a series of dienes, and the catalyst can be reused while maintaining its high activity and selectivity.

**Scheme 20:**
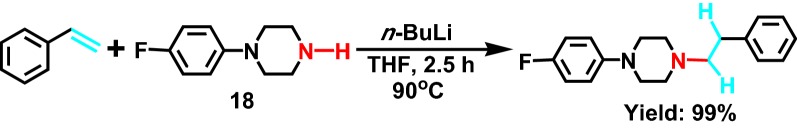
Base-catalyzed hydroamination of styrene with l-(4-fluorophenyl)piperazine

Piperazine derivatives have attracted much attention of chemists because of its very good pharmic and biological activity. As early as 1998, Belier and Breind [34] found that in the *n*-BuLi/THF system and in the absence of any catalyst and additives, they also achieved intermolecular hydroamination reaction between styrene and piperazine compound **18** (Scheme 20), and this reaction can generate a single *anti*-Markovnikov product with a high yield. However, *n*-butyl lithium has a large limitation and can be used only for piperazine compounds.

**Scheme 21:**
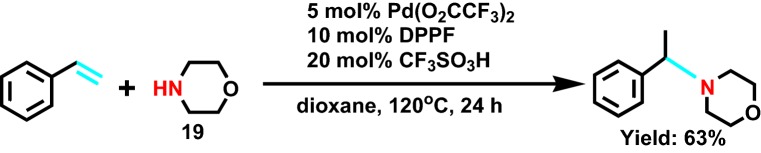
Pd-catalyzed hydroamination of alkylamines with vinylarenes

In 2003, Utsunomiya et al [35] reported the synthesis of morpholine derivatives with Tf-OH and palladium salt. From the view of thermodynamics, in the effect of palladium salt, the reaction formed *η* 3-styrene transition state was more easily than the *η* 3-alkyl transition state, then the intermediate state removed Tf-OH by hydroamination with **19** (Scheme 21), further generating markovnikov products.

**Scheme 22:**
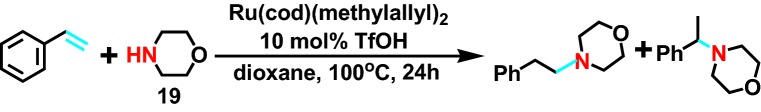
Ruthenium-catalyzed hydroamination of vinylarenes with alkylamines

In second years, Utsunomiya et al [36] improved the catalytic system and used the ruthenium complex to catalyze the synthesis of morpholine derivatives, based on the substrate **19** (Scheme 22) likewise. From the kinetic point of view, this reaction is conducive to the formation of *anti*-Markovnikov amine ruthenium intermediate. In the presence of trifluoromethanesulfonic acid, the rapid irreversible deprotonation reaction occurs in the middle of the anti-Markovnikov amine ruthenium, and then occurring the elimination of beta hydrogen to get the *anti*-Markovnikov additive product.

### Unsaturated fatty amine

The hydroamination of unsaturated fatty amines as substrates has been studied for decades [37]. These substrates are often concentrated in the imidazole, pyrrole and other nitrogenous heterocyclic compounds [38]. Based on our research [39–42], it is noteworthy that such substances are very important intermediates for synthetic drugs and natural products.

**Scheme 23:**
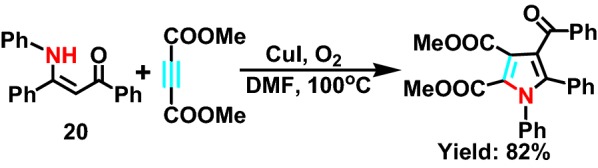
Synthesis of polysubstituted pyrroles from dialkyl ethylenecarboxylate and *β*-enamino ester

In 2010, the Yan project group [43] reported a new type of intermolecular hydroamination of unsaturated fat secondary amines catalyzed by copper. Among them, CuI as a key catalyst, and oxygen as an oxidant, they provide highly selective pyrrole compounds. And the author provides a preliminary mechanism to experience the catalytic cycle of Cu(I/II). The reaction activates secondary amine **20** (Scheme 23) under the action of CuI, and then isomerization occurs in the intermediate state at high temperature. After that, pyrrole compounds are produced by [3 + 3] cyclization, and hydrogen peroxide is released at the same time.

**Scheme 24:**
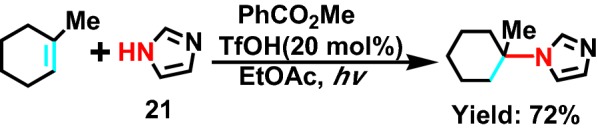
Photoinduced additions of azoles to 1-methyl-1-cyclohexene

In 2008, Moran et al [44] reported that methyl benzoate as photosensitizer, through 254 nm ultraviolet light initiation, imidazoles unsaturated fatty secondary amine **21** (Scheme 24) and a series of olefins were photoisomerized in the process of hydroamination, obtaining complex Markovnikov products. It has been shown that the synthesized series of compounds have antifungal activity.

**Scheme 25:**
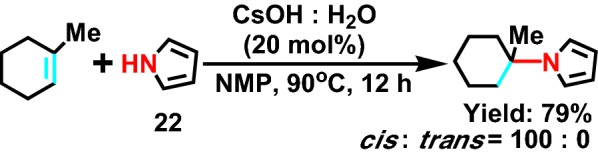
The addition of alcohols and secondary amines by the cesium hydroxide and CsOH catalyzed in NMP

In 1999, the Tzalis group [45] reported that pyrrole was used as a substrate to synthesize a series of different pyrrole derivatives. The author took pyrrole **22** (Scheme 25) as starting materials, with cyclohexene, occurred hydroamination reaction catalyzed by cesium hydroxide monohydrate. This method can also be applied to the synthesis of other nitrogen heterocyclic compounds, such as indole, imidazole, etc.

**Scheme 26:**
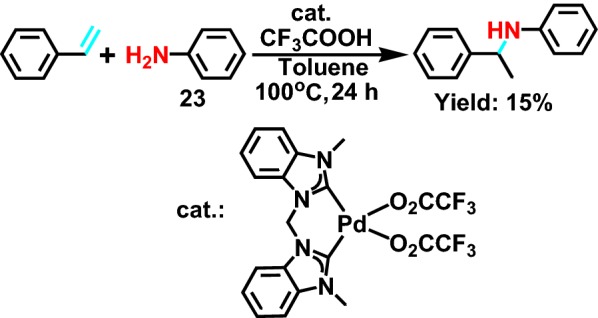
Pd-catalyzed hydroamination of styrene with aniline

In 2009, Huynh et al [46] have presented a straightforward and efficient synthesis of benzannulated dicarbene complexes bearing labile acetato, fluoroacetato, and acetonitrile co-ligands, which are unusually stable in solution and resist ligand disproportionation. The molecular structure of the complexes was determined by X-ray single crystal diffraction. A preliminary catalytic study showed that the reaction between styrene and aniline **23** (Scheme 26) using hydroamination reaction showed the certain activity of complex containing trifluoro ethyl ester.

**Scheme 27:**
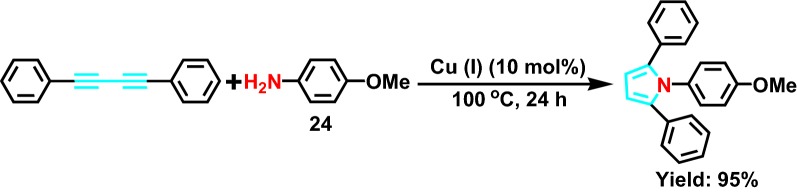
One-pot synthesis of 1, 2, 5-three substituted pyrrole

In 2010, Zheng et al [47] reported a simple synthesis route of 1, 2, 5-three substituted of pyrrole. Under 100°, with CuCl as catalyst, intermolecular and intramolecular double hydroamination reaction has generated between 1,3-butadiyne and primary amine **24** (Scheme 27), 1,4-two substituted 1,3-butadiyne and alkynes through selective intermolecular hydroamination to form 1, 2, 5-three substituted pyrroles with a high yield. And it has the advantages of easy to start, mild reaction conditions, cheap catalyst, and high yield.

**Scheme 28:**
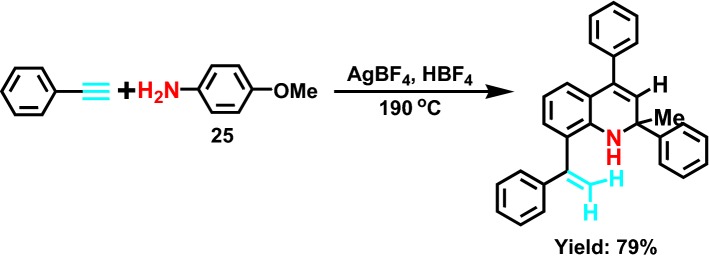
A silver-catalyzed domino reaction of simple aniline and alkyne

In 2005, Luo et al [48] reported a new synthetic method of highly selective multi substituted 1,2-two hydrogen quinoline derivatives under a series of domino **25** (Scheme 28) processes and the catalysis of silver catalyst. Hydrogenation, alkylation, intramolecular hydrogenation and hydrogenation of three molecular alkyl can be completed in the single pot process of the 100% atom economy.

**Scheme 29:**
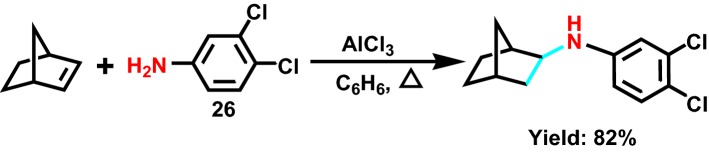
Lewis acid catalyzed hydroamination of norbornene with aromatic amine

In 2008, cheng et al [49] studied the effects of different lewis acids on intermolecular hydroamination by hydroamination of aromatic amine **26** (Scheme 29) with norbornene. The common metal halides and their catalytic properties were compared. BiCl_3_ is the most efficient, delivering a higher yield in a shorter response time. ZrCl_4_ catalytic reaction can be completed at a relatively low temperature, but requires a higher and longer reaction time. Most of the reactions catalyzed by FeCl_3_ have chemical selectivity. When AlCl_3_ is used as a catalyst, some amines can be substituted by different functional groups. The acidity of amine hydrogen atoms is an important factor for conversion benefit.

**Scheme 30:**
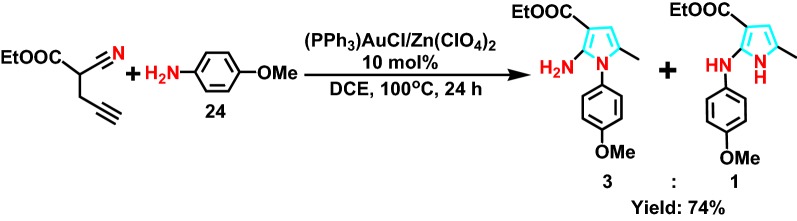
Au(I)/Zn(II)-catalyzed sequential intermolecular hydroamination reaction of 4-yne-nitriles with amine

**Scheme 31:**
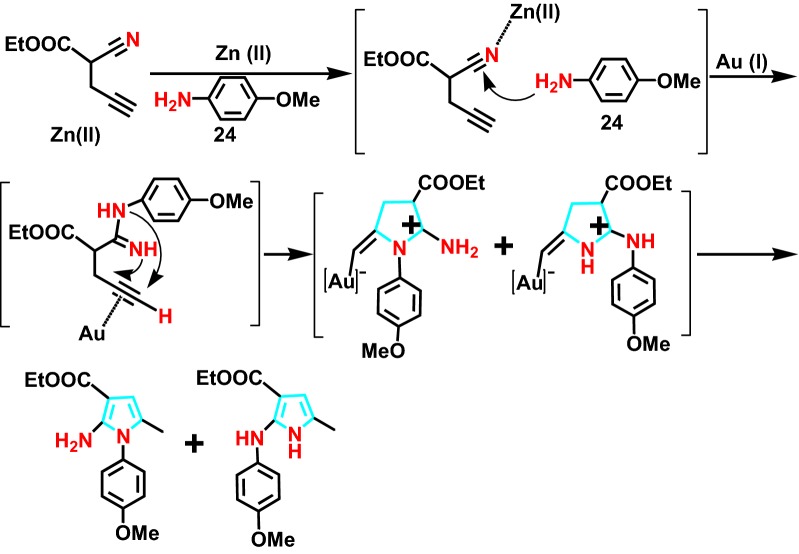
Plausible mechanism for pyrrole formation by Au(I)/Zn(II)-catalyzed

In 2010, Demir et al [50] successfully developed a 4-amine **24** (Scheme 30) cyclochemistry catalyzed by Au(I)/Zn(II) in series as well. An effective, versatile and widely available synthetic pyrrole with multiple substituents is provided. Au (I) species combined with Zn (II) salts to catalyze hydrogenation. The reaction mechanism was further studied as shown in Scheme 31, the product distribution of the reaction was elucidated, and the range of synthesis was expanded.

**Scheme 32:**
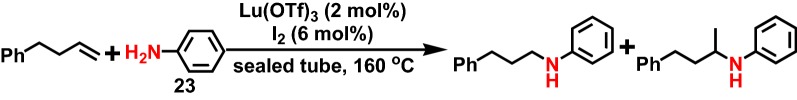
Intermolecular hydroamination of unactivated alkenes and anilines catalyzed by lanthanide salts

In 2009, Yin et al [51] reported that Lu(OTf)_3_/I_2_ catalytic system had better catalytic activity in the hydroamination reaction of inactive olefin and similar aniline **23** (Scheme 32). This system has the advantages of simple use, cheap catalyst, atomic economy and high yield. The proposed catalytic system provides a good strategy for hydroamination under mild conditions.

## Conclusion and outlook

In summary, the raw materials of hydroamination, whether alkyne, alkene, amine or olefin, are widely existed in various moieties, applying for high atomic economy in artificial synthesis [52–54]. Over the past decades, heterogeneous catalysis for a more sustainable hydroamination due to the possibility of recycling and simple isolation of the secondary amines or imines by simple centrifugation or filtration of the solid, avoiding work-up and metal contamination of the product. It is believed that in the near future, hydroamination can replace those unsustainable reactions of methodology during the industrial circles, especial for medicine and paint intermediate, such as coupling reaction and Wittig reaction. In spite of these excellent achievements, research on the use of nonprecious metals is still open for both hydroamination and *C*-*N* formations. Soon intensive work will focus not only on new metal-organic design in the solid state, i.e. metal-organic frameworks or zeolites, also allow the stability, low toxicity and reusability of such heterogeneous catalysts [55].

## Data Availability

Not applicable.
